# Genetic Diversity and Evolutionary Kinetics of Influenza A Virus H3N2 Subtypes Circulating in Riyadh, Saudi Arabia

**DOI:** 10.3390/vaccines11030702

**Published:** 2023-03-20

**Authors:** Gani Asa Dudin, Ibrahim M. Aziz, Rasha M. Alzayed, Anwar Ahmed, Tajamul Hussain, Ali M. Somily, Muslim M. Alsaadi, Fahad N. Almajhdi

**Affiliations:** 1 Department of Botany and Microbiology, College of Science, King Saud University, Riyadh 11451, Saudi Arabia; 2 Biology Department, College of Science, Jouf University, Sakaka 41412, Saudi Arabia; 3 Center of Excellence in Biotechnology Research, College of Science, King Saud University, Riyadh 11451, Saudi Arabia; 4 Department of Pathology, College of Medicine, King Saud University, Riyadh 11451, Saudi Arabia; 5 Department of Pediatrics, College of Medicine, King Saud University, Riyadh 11451, Saudi Arabia

**Keywords:** influenza A virus, Non-H1N1, H3N2, sequence analysis, phylogenetic analysis, vaccine strains

## Abstract

Presence of a large foreign workforce and the annual gathering of people for pilgrimage from around the globe have significantly contributed to the emergence and diversity of respiratory viruses in Saudi Arabia. Here, we report the sequence and phylogenetic analysis of the H3N2 subtype of influenza A virus (IAV) in clinical samples collected from Riyadh, Saudi Arabia. Based on RT-PCR, IAV was found in 88 (28.3%) of the 311 samples screened. Of the 88-IAV positive samples, 43 (48.8%) were H1N1 subtype while the remaining 45 (51.2%) were found to be of the H3N2 subtype. Complete sequencing of HA and NA genes of H3N2 revealed, twelve and nine amino acid (AA) substitutions respectively, and importantly, these variations are absent in the current vaccine strains. Based on the phylogenetic analysis, the majority of H3N2 strains were grouped in the same clades as the vaccine strains. Importantly, the N-glycosylation sites at AA 135(NSS) were found to be unique to 6 strains in the investigated HA1 protein and were absent in the current vaccine strains. These data may have significant clinical implications in designing novel and population-based vaccines for IAV and underscore the need for regular monitoring of efficacy of vaccines due to emerging variants.

## 1. Introduction

Acute respiratory tract infections (ARTIs) are the major public health concerns [1]. Viruses are considered the major cause of ARTIs worldwide. Of all, seven groups of viruses are mostly incriminated as potential pathogens responsible for ARTIs including respiratory syncytial, influenza, parainfluenza, rhino-, metapneumo-, adeno-, and corona- viruses [2,3,4].

Influenza viruses have long been known to cause significant morbidity, mortality, and economic and social burden. Annual seasonal influenza epidemics result in around 1 billion cases of seasonal influenza occurring globally each year; among which 3–5 million suffer from severe illness, 300,000 to 500,000 deaths, and infect up to 20% of the population [5].

Influenza viruses are grouped in the family *Orthomyxoviridae*. Viruses in this family are characterized by having a single-stranded, negative sense, and segmented RNA genome [6]. Several influenzas A subtypes that infect humans were detected in many countries worldwide such as H5N1, H5N2, H7N2, H7N3, H7N7, H9N2, and H10N7. Recently, four additional AIV subtypes, H7N9, H6N1, H10N8, and H5N6, have been reported in humans. These viruses differ in their pathogenicity and symptoms in the patients varying from mild to severe and can be fatal in many cases [7,8].

Hemagglutinin (HA) and neuraminidase (NA) are the two main structural glycoproteins located on the surface of the influenza A virus particle, where, HA is responsible for the attachment of the virus with host cell surface, while NA causes detachment of virus from host cell. Each HA monomer is synthesized as an inactive precursor protein HA0. The cleavage of HA0 into HA1 and HA2 subunits by a cellular protease is a prerequisite for IAV infectivity and is important for pathogenicity in humans [9,10].

Influenza vaccination remains the most effective preventive measure to prevent severe diseases caused by influenza. Monovalent vaccines were the first to be developed after the identification of the H1N1 subtype in humans. Nowadays, two types of IVs are mainly used trivalent influenza vaccines (TIVs) and quadrivalent influenza vaccines (QIV) [11].

Saudi Arabia is one of the major countries at risk of the emergence and evolution of respiratory viruses due to the high population of the foreign workforce and the annual gathering of pilgrims during Hajj [12]. The current epidemiological and phylogenetic of circulating IAVs in Saudi Arabia are seriously deficient, especially for the non-H1N1 subtype. Vaccine strains for the 2022–23 influenza vaccines were recommended by the WHO as QIV for use in the northern hemisphere influenza seasons. The strains of the influenza virus circulating in the population change and evolve over time [13]. The flu viruses in seasonal flu vaccines are reviewed each year by the World Health Organisation (WHO) which makes recommendations on strains of the influenza virus that are to be included in the following year’s vaccine. This study aimed to identify IAV subtypes other than H1N1 that circulate in Riyadh and assess their epidemiological characteristics, genetic diversity, and evolutionary kinetics circulating from (2014–2020) and the efficacies of the available vaccines.

## 2. Materials and Methods

A total of 311 clinical samples involving nasopharyngeal aspirates (NPAs), throat swabs, and sputum were collected from suspected influenza infection patients from emergency rooms and hospital wards at King Khalid University Hospital (KKUH) during the epidemic years 2014/2015, 2015/2016, 2016/2017, 2017/2018, and 2019/2020. Fever, cough, sore throat, runny nose, muscle or body aches, headache, and fatigue were considered to be typical influenza symptoms, according to the protocol of Influenza Surveillance in Saudi Arabia (ISSA) (Saudi MOH, 2017). Immediately after collection, samples were mixed with 2 mL of the virus transport medium and transported under cool conditions to the Virology Research Laboratory, College of Science, King Saud University, and stored at −80 °C until analyzed. This study was approved by Research Ethics Committee (RES) at the King Saud University, Riyadh, Saudi Arabia (Ethics Reference No. 14/4463/IRB 03 December 2014).

### 2.1. Detection, Typing, and Sequencing of IAV

Viral RNA was extracted from clinical samples using QIAamp Viral RNA Extraction Kit (Qiagen, Hilden, Germany) according to the manufacturer’s instructions. IBV was detected with universal primer One-Step Ahead RT-PCR Kit with Taq High Fidelity DNA Polymerase (Qiagen, Hilden, Germany, cat. no. 220213). The reaction was performed in a GeneAmp 9700 thermal cycler using the following cycling conditions: reverse transcription at 50 °C for 30 min; initial denaturation at 95 °C for 15 min; 35 cycles of denaturation at 94 °C for 30 s, annealing at 52 °C for 30 s, and extension at 72 °C for 2 min; and a final extension step of 72 °C for 10 min. The amplified products were visualized in 1% ethidium-bromide stained agarose gel and compared to a 100 bp Plus DNA ladder (Qiagen, Hilden, Germany). Primer sequences used for the detection, typing (H1N1 and H3N2) are listed in Table 1.

The *HA* and *NA* antigenic glycoprotein genes of IAV/H3N2 was the target for amplification. PCR was performed in a GeneAmp 9700 thermal cycler using the same kit. The two overlapping primer sets (H3A1F6 and HA828F) were used to obtain complete HA as well as (NA-1F and NA636F) to obtain complete NA genes. Primer sequences used are listed in Table 1.

The amplified fragments of the HA and NA genes were sequenced in both directions using commercial service (Macrogen Inc, Seoul, South Korea). DNA sequences were edited, and overlapping fragments were assembled using the Bioedit program, version 7.0 (Ibis Biosciences, Carlsbad, CA, USA). The final sequences in this study have been deposited in GenBank with accession numbers presented in the supplementary file (Table S1).

### 2.2. Sequence Data Analysis and Phylogenetic Analysis

The nucleotide sequences were edited, assembled, and aligned using BioEdit Sequence Alignment Editor Ver 7.0.5.2. Divergence analysis, identification of mutation sites, and prediction of AA changes were carried out using EditSeq and MegAlign programs, and Lasergene software (DNAStar Inc., Madison, WI, USA). The sequences of reference strains of known clades and vaccine strains recommended by WHO were obtained from the GenBank and GISAID databases (Table S2). Heterogeneity in the N-glycosylation (Asn-X-Ser/Thr, where X is any amino acid (AA) except proline) and O-glycosylation sites (Ser or Thr) in HA and NA proteins of H3N2 strains were assessed using NetNGlyc 1.0 [16] and NetOGlyc 3.1 [17]. Phylogenetic analysis was performed by the neighbor-joining method using MEGA 11 (v.11, Pennsylvania State University, University Park, PA, USA). The number of bootstrap replications was set to 1000 and bootstrap values above 60% were labeled on major tree branches for reference.

### 2.3. Statistical Analysis

Fisher’s Exact test was used to identify the significant differences in the prevalence of Influenza A virus within the categories of gender and age groups. Post hoc comparison was obtained from Z-test using bonferroni correction. *p* < 0.05 was considered significant.

## 3. Results

### 3.1. Detection of IAV and Subtyping

The five seasonal IAVs (2014–2018 and 2019/2020) were detected in 311 (155 males and 156 females) newly collected and archival clinical NPAs samples using a one-step RT-PCR assay. Among the five studied seasons, the prevalence of IAV (88 out of 311 samples; 28.3%) in all the five studied seasons, and H1N1 strains were more prevalent (45 out of 88 samples; 51.2%) compared to H3N2 strains (43 out of 88 samples; 48.8%). The age groups were divided according to the high-risk population for infection with influenza-like illness (ILI) /acute respiratory illness (ARI) [18,19]. There were 4 segregated groups such as 0–4 years, 5–14 years, 15–64 years, and ≥65 years. The data on the incidence of IAV, H1N1 and H3N2 in different categories are provided in Table 2. Age group 5–14 year have significantly higher prevalence of Influenza A virus as compared to younger group aging 0–4 years (*p* < 0.05) and also elderly aging ≥65 years (*p* < 0.05). Furthermore, those aging 15–64 years also had significantly higher prevalence of Influenza A virus as compared to elderly aging more than 65 years (*p* < 0.05). No significant differences were found between 5–14 and 15–64 years age groups as well as between 0–4 years and 15–64 years age groups.

### 3.2. Sequence Analysis of HA and NA Genes of H3N2 

#### 3.2.1. Nucleotide and AA Sequence Analysis of *HA* Gene of H3N2 Subtype Study Strains

The complete nucleotide sequence of the *HA* gene of 22 H3N2 study strains (1701 nucleotides) was sequenced and aligned with sequences of 50 globally circulating in the GISAID database (Table S2) using the Clustal W method. A total of 66 mutated nucleotide sites (3.24%) were found in *HA* gene of H3N2, of which only 37 mutation sites (33.64%) could lead to AA changes, ten of these (10.15%) were unique and not recorded before and considered unique mutations as compared with 50 representatives of currently circulating strains. 

#### 3.2.2. Sequence Analysis of *NA* Gene of H3N2 Strains

In the *NA* gene sequence of 22 H3N2 strains, the nucleotide of full-length *NA* gene sequences was 1410 bp, a total of 67–77 nucleotide mutation sites (4.75–5.46%) were detected, and 27 (35.06%) of the 77 polymorphic sites were found that lead to AA change, and the 7 AA substitution sites of these were recorded in reference strains as compared to currently H3N2 circulating strains available in the GISAID database (Table S2). 

**Figure 1:**
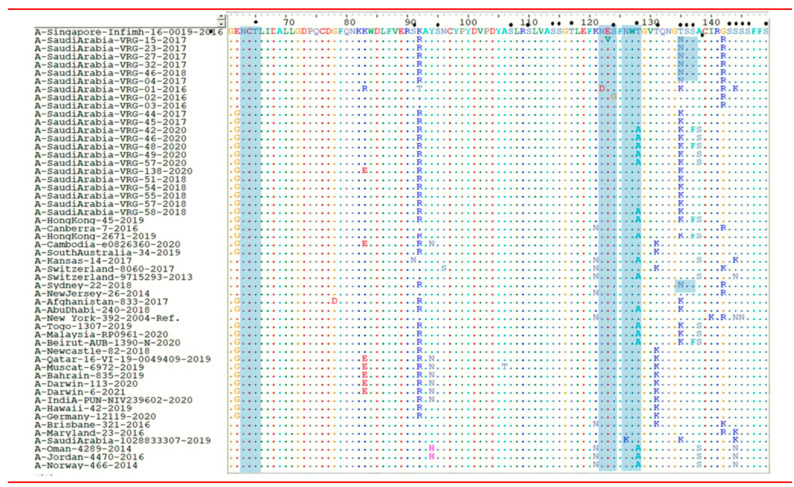
Alignment of the HA1 domain of H3N2 strains. The AAs were aligned to the sequences of the current vaccine (Influenza A/Singapore/infimh-16-0019/2016(H3N2)-like virus. The coloured alphabets indicate amino acid changes, identical residues are presented by dots. The predicted N-glycosylation sites are enclosed in blue rectangles. Small filled circles correspond to the predicted O-glycosylation sites.

The mutations in the HA1 domain in Riyadh strains were further examined and compared with the current vaccine strains of H3N2 viruses (A/Singapore/infimh-16-0019/2016(H3N2), A total of 12 mutated nucleotide sites were found in *HA* gene of H3N2, 7 of these were unique and not recorded before as compared with currently circulating vaccine strains as shown in (Table 3 and Figure 1).

We performed the same analysis in the *NA* gene of H3N2 strains. Most of the study strains had 9 AA substitutions in the *NA* gene that were unique and not recorded in the current vaccine strains of H3N2 viruses (A/Singapore/infimh-16-0019/2016(H3N2), the vaccine provided by the Saudi Ministry of Health [20] as shown in (Table 4) and Figure 1.

### 3.3. N- and O-Glycosylation Site Analysis in the AA Sequence

The number of N-glycosylation sites in the investigated open reading frame of HA1 protein of H3N2 varied from 8 to 9. All the potential N-glycosylation sites were reported in vaccine strains and were also found in the strains from this study. In addition, the AA substitution T at position 135 into N (T135N) gave rise to an additional potential N-glycosylation site 135(NSS) in 6 strains (A-SaudiArabia-VRG-04-2017, A-Saudi Arabia-VRG-15-2017, A-Saudi Arabia-VRG-23-2017, A-Saudi Arabia-VRG-27-2017, A-Saudi Arabia-VRG-32-2017, and A-Saudi Arabia-VRG-46-2018) were unique and not recorded in the current vaccine strains. Conversely, the HA1 domain is heavily glycosylated with O-linked carbohydrates at serine and threonine residues. The number of potential O-linked glycosylation sites generally ranging from 46 to 55 were reported A/Singapore/Infimh-16-0019/2016, and also found in AA of all strains from this study as shown in Figure 1.

### 3.4. Phylogenetic Analysis 

**Figure 2:**
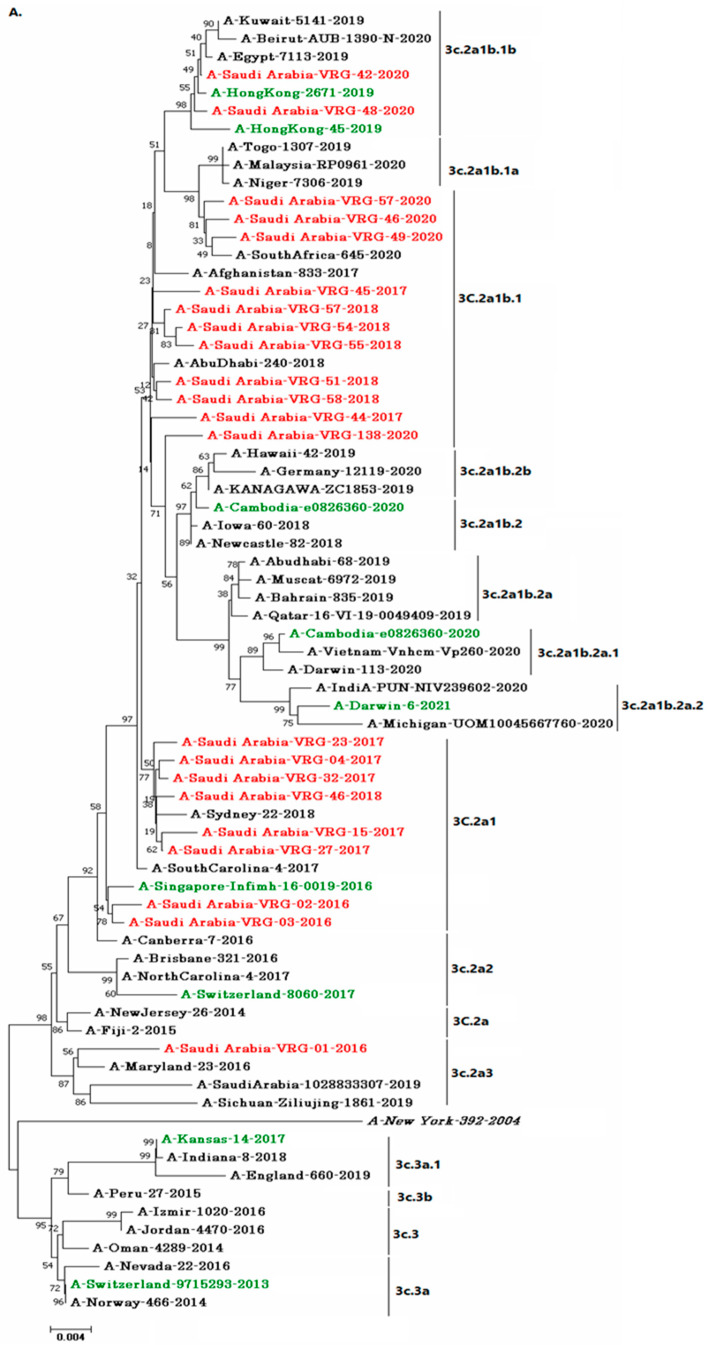

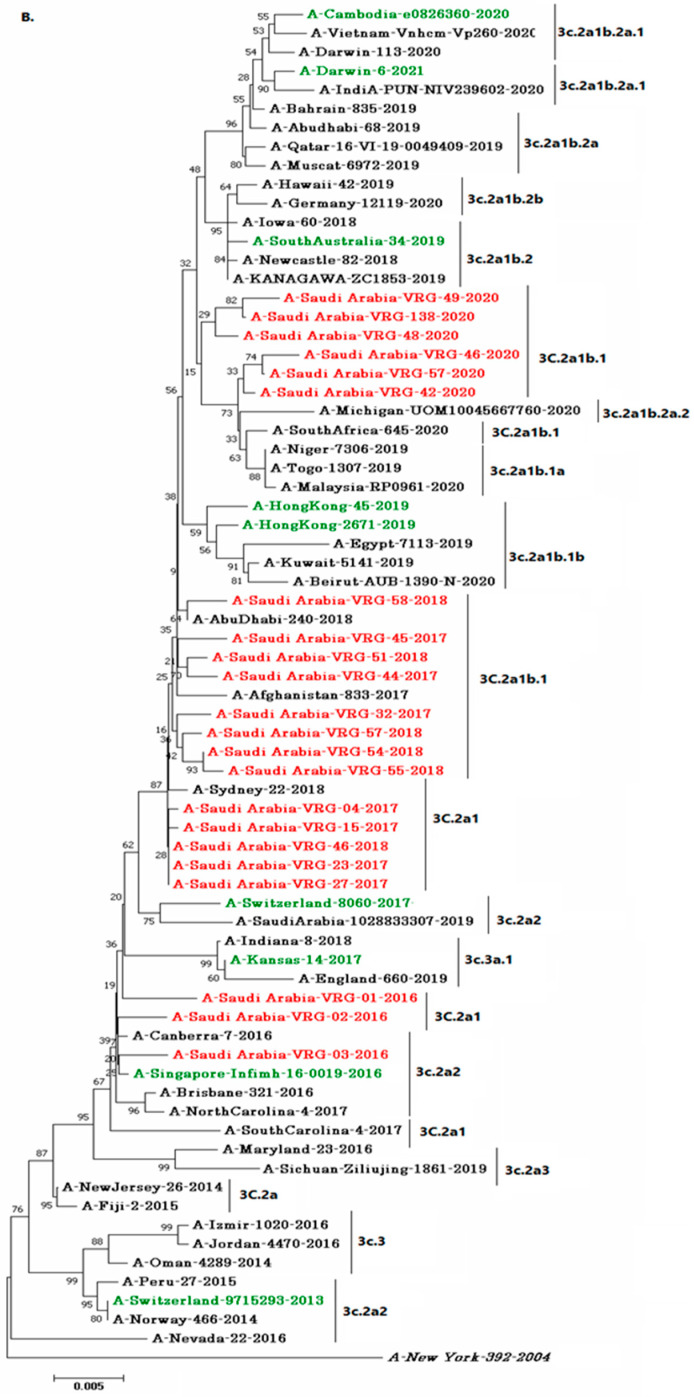
Phylogenetic for H3N2 strains, according to *HA* gene (**A**), *NA* gene (**B**). The Saudi H3N2 strains are indicated with red ink whereas the vaccine strain is indicated using green ink. The reference strains are denoted by black and italics.

Phylogenetic analysis of the HA and NA sequences of the H3N2 strains showed 22 of them were grouped in 4 clusters with the majority of them were grouped under the 3c.2a1b.1 (A-Saudi Arabia-VRG-46-2020, A-Saudi Arabia-VRG-49-2020, A-SaudiArabia-VRG-57-2020, A-Saudi Arabia-VRG-45-2017, A-Saudi Arabia-VRG-51-2018, A-Saudi Arabia-VRG-54-2018, A-SaudiArabia-VRG-55-2018, A-Saudi Arabia-VRG-57-2018, A-SaudiArabia-VRG-58-2018, A-Saudi Arabia-VRG-44-2017, and A- SaudiArabia-VRG-138-2020) meanwhile 8 of them were categorized under the 3c.2a1 cluster (A-Saudi Arabia-VRG-04-2017, A-Saudi Arabia-VRG-23-2017, A-Saudi Arabia-VRG-32-2017, A-Saudi Arabia-VRG-46-2018, A-Saudi Arabia-VRG-15-2017, A-Saudi Arabia-VRG-27-2017, A-Saudi Arabia-VRG-02-2016, and A-Saudi Arabia-VRG-03-2016 with a sequence homology of 58% with the vaccine strain A/Singapore/Infimh-16-0019/2016 as shown in Figure 2.

## 4. Discussion

Saudi Arabia is one of the major countries with a continued risk of the emergence and evolution of respiratory viruses due to large influx of foreign workforce and the annual religious gathering of people from all over the world. Although, previous studies from Saudi Arabia have reported the subtypes responsible for IAV infections, such information involving the circulating H3N2 subtype in Riyadh in spatial temporal basis are absent. Therefore, the current study aimed to identify IAV subtypes other than (H1N1) and assess their epidemiological characteristics, genetic diversity, and evolutionary kinetics circulating from 2014 to 2020 and inform policy decisions on influenza vaccines. 

In the present study, IAV was identified in 88 (28.3%) of the 311 clinical samples 2014–18 and 2019/20) in Riyadh., Of the 88 IAV positive samples, 45 (51.2%) were caused by H1N1 infections, whereas the remaining 43 (48.8%) were of H3N2. These observations are consistent with a previous study 15 countries in the Eastern Mediterranean Region, where H1N1pdm09 subtype was frequently circulated subtype with 1666 cases (58.5%), followed by H3N2 with 671 cases (23.6%) [21]. In contrast, the H3N2 strains were found to be more common in western Saudi Arabia with (42.0%) than H1N1 pdm09 (27.3%) during October 2015 to 2019 [22]. The H3N2 strains were also reported to be more predominance with 18 (72%) cases than H1N1 with 7 (28%) at the Hajj gatherings during 2013 to 2015 (27.3%) during October 2015 to 2019 [23].

In the present study, we also found that H3N2 strains were associated with the higher rates of infections in females (28.8%) than in males (27.7%). These observations are in parallel with an earlier study from the western Saudi Arabia that reported that H3N2 strains were more frequent in females (51.0%) among adult patients (19–60) years old [22]. H3N2 strains were also more frequent in females (55.0%) among adult patients (19–60) years old [24]. Furthermore, nearly half of the influenza-related deaths in the 1918 pandemic were in young adults 20–40 years of age [25].

Vaccination is the most effective way to prevent influenza virus infection and its complications. Influenza vaccines have been available since 70 years ago and are reformulated over time [26,27]. The recommendation is that 6 months of age and older must receive the influenza vaccination annually, unless contraindicated [28]. The majority of influenza vaccines used worldwide are TIVs containing two IAV (H3N2 and H1N1) and only one IBV strain (B/Victoria or B/Yamagata) [29]. Recently, influenza vaccines are mainly QIV containing two IAV (H3N2 and H1N1) and two IBV strains) and most of them are manufactured by growing influenza viruses using eggs in a traditional manner [30]. The vaccine provided by the Saudi Ministry of Health was a trivalent inactivated influenza virus vaccine with the following strains: Influenza A/Michigan/45/2015(H1N1) pdm-09 virus, Influenza A/Singapore/INFIMH-16-0019/2016(H3N2)-like virus, and Influenza B/Colorado/06/2017-like virus (B/Victoria/2/87 lineage) [20]. WHO has managed to identify and recommend more than 28 vaccine strains [31]. In this study, nine H3N2 vaccine strains representing the north hemisphere were used as references. The comparison of AA substitution between the Saudi H3N2 and vaccine strains was expected to give a prediction as to whether the existing vaccines are still effective against the circulating H3N2 strain in Riyadh, Saudi Arabia. In the current study, there were twelve mutation sites in the HA1 domain and nine AA substitutions in the *NA* gene that were unique and not recorded in the current vaccine strains of H3N2 viruses (A/Singapore/infimh-16-0019/2016(H3N2).

This study also revealed that all study H3N2 strains were in the root clade 3c.2a, and were able to be further separated into subclades according to the AA changes in the domain HA1. VRG-01/2016 was found in the clade 3.2a3 with three other strains (A/Maryland/23/2016, A/Saudi Arabia/1028833307/2019, and A/Sichuan-Ziliujing/1861/2019). A/Saudi Arabia/VRG-02/2016, A/Saudi Arabia/VRG-03/2016, were in the root 3c.2a together with A/New Jersey/26/2014, A/Fiji/2/2015, and A/Canberra/7/2016. While 13 other strains were in the subclade 3C.2a1b, they were characterized by mutations of F159Y, K160T, N171K, N121K, K92R, and H311Q [32,33], and 16 strains were grouped in the 3c. clades/subclade; Clade 3c.2a consisted of two members (A/Saudi Arabia/VRG-2/2016 and A/Saudi Arabia/VRG-7/2016) which were marked by mutations F159Y and K160T [34,35]. The studied strains were still in the same clade as the vaccine strains, with the exception of A/Saudi Arabia/VRG-01/2016, which was in the clade 3c.2a3, where no vaccine stains were located. Clustering in the NA phylogenetic tree was seen similar to the HA tree. However, phylogenetic analyses revealed that of the IAV strains did not always cluster fall within the regional isolates, thus, implying that travel played a role in global distribution. This is also why, in recent years, the vaccine recommendations for the northern and southern hemispheres were nearly identical. 

The HA and NA genes undergo continuous genetic changes, which enable the virus to escape host immune responses and result in influenza seasonal epidemics [36]. Glycosylation of HA is important for the folding and stability of the protein and, in some cases, significantly affects receptor binding and cleavage of the precursor HA0 protein, which in turn influences the virulence and antigenicity of the virus [37]. The putative N-linked glycosylation sites on the HA1 domain has been associated with viral immune evasion [38]. We found 8 to 9 N-glycosylation sites in the HA1 protein of H3N2 and also in the vaccine strains. The N-glycosylation sites at AA 135(NSS), was unique to 6 strains from this study were unique and was not recorded in the current vaccine strains. These findings are in line with an earlier study from Yokohama, Japan carried out during 2016/17 and 2017/18, which reported that some viruses in the clade 3C.2A1b possessed the T135K or T135N substitution causing the loss of the glycosylation site at positions 133–135 (NGK or NGN) but the shift of the glycosylation site at positions 135–137 (NSS) [39]. Similar observations were also found in Quzhou, during 2015–2017 [40]. Further, another study reported that some of the T135N mutations also created new glycosylation sites at N135.

Thus, H3N2 strains that possess HA1 domains with different N-linked glycosylation sites, might have co-circulated and with altered antigenicity [38]. In general, the AA mutations of the study strains are not significant compared to that of the vaccine strains except for positions T135N and G142R. There were 5 and 9 studied strains carried the respective mutations when aligned and compared to that of the vaccine strains.

The limitations of this study were its cross-sectional nature and small sample size. Further, the study cannot suggest any cause for recurrent infections. Additional comprehensive investigations with a larger sample size covering different regions of Saudi Arabia in consecutive epidemic seasons are warranted for a more comprehensive understanding of IAV(H3N2) circulation.

## 5. Conclusions

In conclusion, we report here the genetic diversity in H3N2 subtype isolated in Riyadh in 2014/2018 to 2019/2020 due to the *HA*, and *NA* gene mutations and their influence on the respective proteins in clinical sample. This study showed the prevalence of IAV (88 out of 311 samples; 28.3%) in all the five studied seasons, and H1N1 strains were more prevalent with 51.2% compared to H3N2 strains with 48.8%. H3N2 strains were more frequent in females (28.8%) than in males (27.7%). Twelve mutation sites in the HA1 domain and nine AA substitutions in the *NA* gene of H3N2 strains found in this study were not recorded in the current vaccine strains of H3N2 viruses. The AA substitution at position 135 (T135N) gave rise to an additional potential N-glycosylation site 135(NSS) in 6 strains found in this study are also not part of the current vaccine strains. This may have aided the virus to evade host immune responses and contributed to recurrent infections. Finally, the present findings underscore the need for regular monitoring of the efficacy of vaccines due to emerging variants.

## Figures and Tables

**Table 1:** List of IAV for the detection, typing (H1N1 and H3N2), and sequencing primers included in this study.

Primer Description	Type/Subtype	Gene	Primer Name	Sequence 5′-3′	Product Size (bp)	Ref.
Primers used for detection	IAV		M30F2/08	ATGAGYCTTYTAACCGAGGTCGAAACG	244	[14]
			M264R3/08	TGGACAAANCGTCTACGCTGCAG		
Primers used for typing	(H1N1) Pdm09		HKU-SWF	TGAGCTCAGTGTCATCATTTGA	174	[14]
			HKU-SWR	TGCTGAGCTTTGGGTATGAA		
	H3N2		H3A1F6	AAGCAGGGGATAATTCTATTAACC	1127	
			H3A1R1	GTCTATCATTCCCTCCCAACCATT		
Primers used for sequencing	H3N2	HA	H3A1F6	AAGCAGGGGATAATTCTATTAACC	1127	[14]
			H3A1R1	GTCTATCATTCCCTCCCAACCATT		
			HA828F	ACGAAGTGGGAAAAGCTCAATA	934	[15]
			HA1778R	AGTAGAAACAAGGGTGTTTT		
	H3N2	NA	NA-1F	GAGCAAAAGCAGGAGTAAAG	807	
			NA787R	TGACAATGTGCTAGTATGAAC		
			NA636F	AGATAGTGTTGTTTCATGGTC	830	
			NA1413Rn	AGTAGAAACAAGGAGTTTTT		

**Table 2:** Distribution of samples within epidemic seasons, gender, and age groups.

		No. of Samples N (%)	Positive for Influenza A Virus N (%)	Positive for	
				H1N1, N (%)	H3N2, N (%)
Total		311	88 (28.3)	45 (51.2)	43 (48.8)
Season	2014–2015	24 (7.7)	-	-	-
	2015–2016	14 (4.5)	14 (100)	11 (78.5)	3 (21.5)
	2016–2017	42 (13.5)	42 (100)	22 (52.4)	20 (47.6)
	2017–2018	15 (4.8)	15 (100)	1 (6.7)	14 (93.3)
	2019–2020	216 (69.5)	17 (7.9)	11 (64.7)	6 (35.3)
Gender	Male	155 (49.8)	43 (27.7)	23 (53.5)	20 (46.5)
	Female	156 (50.2)	45 (28.8)	22 (48.9)	23 (51.1)
Age (Y)	0–4	35 (11.3)	8 (22.9)	8 (100)	-
	5–14	8 (2.6)	6 (75) ^a^	4 (66.6)	2 (33.4)
	15–64	200 (64.3)	65 (32.5) ^b^	30 (46.2)	35 (53.8)
	≥65	68 (21.8)	9 (13.2)	3 (33.3)	6 (66.7)

^a^ Significantly different (*p* < 0.05) compareed to 0–4 and ≥65 years age groups; ^b^ Significantly different (*p* < 0.05) compared to ≥65 years age group.

**Table 3:** Amino acid changes in the HA1 domain of Saudi H3N2 strains in comparison with the vaccine strain.

No.	1	2	3	4	5	6	7	8	9
Mutation sites	5	126	212	220	303	329	344	463	468
A-Singapore-Infimh-16-0019-2016	Q	P	I	K	V	N	E	D	H
A-SaudiArabia-VRG-42-2020	Q	L	V	N	I	S	K	D	P
A-SaudiArabia-VRG-46-2020	K	L	V	N	I	S	K	N	P
A-SaudiArabia-VRG-48-2020	K	L	V	N	I	S	K	N	P
A-SaudiArabia-VRG-49-2020	K	L	V	N	I	S	K	N	P
A-SaudiArabia-VRG-57-2020	K	L	V	N	I	S	K	D	P
A-SaudiArabia-VRG-138-2020	K	L	V	N	I	S	K	N	P
A-SaudiArabia-VRG-46-2018	Q	P	V	N	I	S	E	D	H
A-SaudiArabia-VRG-51-2018	Q	L	V	N	I	S	E	D	H
A-SaudiArabia-VRG-54-2018	Q	L	V	N	I	S	E	D	H
A-SaudiArabia-VRG-55-2018	Q	L	V	N	I	S	E	D	H
A-SaudiArabia-VRG-57-2018	Q	L	V	N	I	S	E	D	H
A-SaudiArabia-VRG-58-2018	Q	L	V	N	I	S	E	D	H
A-SaudiArabia-VRG-04-2017	Q	P	V	N	I	S	E	D	H
A-SaudiArabia-VRG-15-2017	Q	P	V	N	I	S	E	D	H
A-SaudiArabia-VRG-23-2017	Q	P	V	N	I	S	E	D	H
A-SaudiArabia-VRG-27-2017	Q	P	V	N	I	S	E	D	H
A-SaudiArabia-VRG-32-2017	Q	P	A	N	I	S	E	D	H
A-SaudiArabia-VRG-44-2017	Q	L	V	N	I	S	E	D	H
A-SaudiArabia-VRG-45-2017	Q	L	V	N	I	S	E	D	H
A-SaudiArabia-VRG-01-2016	Q	P	V	K	V	T	E	D	H
A-SaudiArabia-VRG-02-2016	Q	P	V	K	V	N	E	D	H
A-SaudiArabia-VRG-03-2016	Q	P	V	K	V	N	E	D	H

Note: red alphabets: sites where amino acid changes occur.

**Table 4:** Amino acid changes in the NA of Saudi H3N2 strains in comparison to the vaccine strain.

No.	1	2	3	4	5	6	7	8	9
Mutation sites	5	126	212	220	303	329	344	463	468
A-Singapore-Infimh-16-0019-2016	Q	P	I	K	V	N	E	D	H
A-SaudiArabia-VRG-42-2020	Q	L	V	N	I	S	K	D	P
A-SaudiArabia-VRG-46-2020	K	L	V	N	I	S	K	N	P
A-SaudiArabia-VRG-48-2020	K	L	V	N	I	S	K	N	P
A-SaudiArabia-VRG-49-2020	K	L	V	N	I	S	K	N	P
A-SaudiArabia-VRG-57-2020	K	L	V	N	I	S	K	D	P
A-SaudiArabia-VRG-138-2020	K	L	V	N	I	S	K	N	P
A-SaudiArabia-VRG-46-2018	Q	P	V	N	I	S	E	D	H
A-SaudiArabia-VRG-51-2018	Q	L	V	N	I	S	E	D	H
A-SaudiArabia-VRG-54-2018	Q	L	V	N	I	S	E	D	H
A-SaudiArabia-VRG-55-2018	Q	L	V	N	I	S	E	D	H
A-SaudiArabia-VRG-57-2018	Q	L	V	N	I	S	E	D	H
A-SaudiArabia-VRG-58-2018	Q	L	V	N	I	S	E	D	H
A-SaudiArabia-VRG-04-2017	Q	P	V	N	I	S	E	D	H
A-SaudiArabia-VRG-15-2017	Q	P	V	N	I	S	E	D	H
A-SaudiArabia-VRG-23-2017	Q	P	V	N	I	S	E	D	H
A-SaudiArabia-VRG-27-2017	Q	P	V	N	I	S	E	D	H
A-SaudiArabia-VRG-32-2017	Q	P	A	N	I	S	E	D	H
A-SaudiArabia-VRG-44-2017	Q	L	V	N	I	S	E	D	H
A-SaudiArabia-VRG-45-2017	Q	L	V	N	I	S	E	D	H
A-SaudiArabia-VRG-01-2016	Q	P	V	K	V	T	E	D	H
A-SaudiArabia-VRG-02-2016	Q	P	V	K	V	N	E	D	H
A-SaudiArabia-VRG-03-2016	Q	P	V	K	V	N	E	D	H

Note: red alphabets: sites where amino acid changes occur.

## Data Availability

All the data is provided in the manuscript.

## Supplementary Materials

The following supporting information can be downloaded at: https://www.mdpi.com/article/10.3390/vaccines11030702/s1, Table S1: List of sequences data submitted to the GenBank with accession numbers; Table S2: List of H3N2 strains included in sequence and phylogenetic analysis.
