# Supplementary material for: Genetic Diversity and Evolutionary Kinetics of Influenza A Virus H3N2 Subtypes Circulating in Riyadh, Saudi Arabia
Source: Vaccines (Basel). 2023 Mar 20;11(3):702. doi: 10.3390/vaccines11030702 (PMC10054866; doi:10.3390/vaccines11030702)
Supplement: Supplementary file 1 [file vaccines-11-00702-s001.zip › vaccines-2248119-Supplementary Materials.pdf]

Table S1: List of sequences data submitted to the GenBank with accession numbers

NO	Strain	Accesion No.		Year
		HA Genes	NA Genes	
1	A/Saudi Arabia/VRG-01/2016(H3N2)	ON514565	ON514227	2016
2	A/Saudi Arabia/VRG-02/2016(H3N2)	ON514617	ON514616	2016
3	A/Saudi Arabia/VRG-03/2016(H3N2)	ON520897	ON520901	2016
4	A/Saudi Arabia/VRG-04/2017(H3N2)	ON521141	ON521167	2017
5	A/Saudi Arabia/VRG-15/2017(H3N2)	ON521169	ON521191	2017
6	A/Saudi Arabia/VRG-23/2017(H3N2)	ON521222	ON521223	2017
7	A/Saudi Arabia/VRG-27/2017(H3N2)	ON522003	ON524169	2017
8	A/Saudi Arabia/VRG-32/2017(H3N2)	ON524170	ON524408	2017
9	A/Saudi Arabia/VRG-44/2017(H3N2)	ON627717	ON524821	2017
10	A/Saudi Arabia/VRG-45/2017(H3N2)	ON524823	ON524824	2017
11	A/Saudi Arabia/VRG-46/2018(H3N2)	ON524839	ON524860	2018
12	A/Saudi Arabia/VRG-51/2018(H3N2)	ON524871	ON525112	2018
13	A/Saudi Arabia/VRG-54/2018(H3N2)	ON527514	ON527515	2018
14	A/Saudi Arabia/VRG-55/2018(H3N2)	ON527517	ON527519	2018
15	A/Saudi Arabia/VRG-57/2018(H3N2)	ON527525	ON527526	2018
16	A/Saudi Arabia/VRG-58/2018(H3N2)	ON527530	ON527548	2018
17	A/Saudi Arabia/VRG-42/2020(H3N2)	OP536171	OP536586	2020
18	A/Saudi Arabia/VRG-46/2020(H3N2)	OP536200	OP536588	2020
19	A/Saudi Arabia/VRG-48/2020(H3N2)	OP536201	OP536589	2020
20	A/Saudi Arabia/VRG-49/2020(H3N2)	OP536205	OP536988	2020
21	A/Saudi Arabia/VRG-57/2020(H3N2)	OP536209	OP537019	2020
22	A/Saudi Arabia/VRG-138/2020(H3N2)	OP536417	OP537028	2020

Table S2: List of H3N2 strains included in sequence and phylogenetic analysis

No.	Strain	Origin	Collection Date	Gisaid Accession No.		Gisaid Clade
				HA	NA	
1.	A-New York-392-2004	USA-Ref.	2004	EPI252225	EPI79013	3C.2a
2.	A-NewJersey-26-2014	USA	2014	EPI841441	EPI841440	3C.2a

3.	A-Fiji-2-2015	New Zealand	2015	EPI636655	EPI636655	3C.2a
4.	A-Canberra-7-2016	Australia	2016	EPI866694	EPI793167	3C.2a1
5.	A-Singapore-Infimh-16-0019-2016	Singapore	2015	EPI1381186	EPI1381185	3C.2a1
6.	A-SouthCarolina-4-2017	USA	2017	EPI1094351	EPI1094350	3C.2a1
7.	A-Sydney-22-2018	Australia	2018	EPI1251865	EPI1251864	3C.2a1b.1
8.	A-Afghanistan-833-2017	Afghanistan	2017	EPI1197229	EPI1197228	3C.2a1b.1
9.	A-AbuDhabi-240-2018	UAE	2018	EPI1245538	EPI1245537	3C.2a1b.1
10.	A-SouthAfrica-645-2020	South Africa	2020	EPI1759254	EPI1759253	3c.2a1b.1a
11.	A-Niger-7306-2019	Niger	2019	EPI1651919	EPI1651918	3c.2a1b.1a
12.	A-Togo-1307-2019	Togo	2019	EPI1683617	EPI1683616	3c.2a1b.1a
13.	A-Malaysia-RP0961-2020	MalaysiA	2020	EPI1759278	EPI1759277	3c.2a1b.1b
14.	A-HongKong-45-2019	China	2019	EPI1691930	EPI1691929	3c.2a1b.1b
15.	A-HongKong-2671-2019	China	2019	EPI1592035	EPI1592034	3c.2a1b.1b
16.	A-Egypt-7113-2019	Egypt	2019	EPI1882614	EPI1882615	3c.2a1b.1b
17.	A-Kuwait-5141-2019	Kuwait	2019	EPI1719377	EPI1719376	3c.2a1b.1b
18.	A-Beirut-AUB-1390-N-2020	Lebanon	2020	EPI1814744	EPI1814743	3c.2a1b.2
19.	A-Iowa-60-2018	USA	2018	EPI1324969	EPI1359998	3c.2a1b.2
20.	A-Newcastle-82-2018	UK	2018	EPI1595444	EPI1595443	3c.2a1b.2
21.	A-SouthAustralia-34-2019	Australia	2019	EPI1607117	EPI1607116	3c.2a1b.2a
22.	A-Qatar-16-VI-19-0049409-2019	Qatar	2019	EPI1619516	EPI1619517	3c.2a1b.2a
23.	A-Abudhabi-68-2019	UAE	2019	EPI1636325	EPI1636324	3c.2a1b.2a
24.	A-Muscat-6972-2019	Oman	2019	EPI1646754	EPI1646900	3c.2a1b.2a
25.	A-Bahrain-835-2019	Bahrain	2019	EPI1696676	EPI1696675	3c.2a1b.2a.1
26.	A-Cambodia-e0826360-2020	CambodiA	2020	EPI1843589	EPI1843588	3c.2a1b.2a.1
27.	A-Darwin-113-2020	Australia	2020	EPI1733843	EPI1733842	3c.2a1b.2a.1
28.	A-Vietnam-Vnhcm-Vp260-2020	Vietnam	2020	EPI1848008	EPI1848007	3c.2a1b.2a.2

29.	A-Darwin-6-2021	Australia	2021	EPI1885402	EPI1885401	3c.2a1b.2a.2
30.	A-Michigan-UOM10045667760-2020	USA	2020	EPI2095215	EPI2095213	3c.2a1b.2a.2
31.	A-IndiA-PUN-NIV239602-2020	IndiA	2020	EPI1843873	EPI1843872	3c.2a1b.2b
32.	A-KANAGAWA-ZC1853-2019	Japan	2019	EPI1398386	EPI1398385	3c.2a1b.2b
33.	A-Hawaii-42-2019	USA	2019	EPI1486401	EPI1486400	3c.2a1b.2b
34.	A-Germany-12119-2020	Germany	2020	EPI1756862	EPI1756861	3c.2a2
35.	A-Brisbane-321-2016	Australia	2016	EPI919282	EPI919281	3c.2a2
36.	A-NorthCarolina-4-2017	USA	2017	EPI925625	EPI925624	3c.2a2
37.	A-Switzerland-8060-2017	Switzerland	2017	EPI1326015	EPI1326014	3c.2a3
38.	A-Maryland-23-2016	USA	2016	EPI868424	EPI868423	3c.2a3
39.	A-SaudiArabia-1028833307-2019	Saudi Arabia	2019	EPI1754145	EPI1754146	3c.2a3
40.	A-Sichuan-Ziliujing-1861-2019	China	2019	EPI1648634	EPI1648633	3c.3
41.	A-Oman-4289-2014	Oman	2014	EPI551900	EPI551901	3c.3
42.	A-Izmir-1020-2016	Turkiye	2016	EPI829311	EPI829312	3c.3
43.	A-Jordan-4470-2016	Jordan	2016	EPI769557	EPI769558	3c.3a
44.	A-Switzerland-9715293-2013	Switzerland	2013	EPI814528	EPI814527	3c.3a
45.	A-Norway-466-2014	Norway	2014	EPI530647	EPI530648	3c.3a
46.	A-Peru-27-2015	Peru	2015	EPI629582	EPI629581	3c.3a
47.	A-Nevada-22-2016	USA	2016	EPI747840	EPI747839	3c.3a.1
48.	A-Kansas-14-2017	USA	2017	EPI1504535	EPI1504534	3c.3a.1
49.	A-Indiana-8-2018	USA	2018	EPI1197165	EPI1197164	3c.3a.1
50.	A-England-660-2019	UK	2019	EPI1741059	EPI1741058	3C.2a
